# Determining sea-level rise in the Caribbean: A shift from temperature to mass control

**DOI:** 10.1038/s41598-024-60201-8

**Published:** 2024-05-06

**Authors:** Deron O. Maitland, Kristin Richter, Roshin P. Raj, Antonio Bonaduce, Kerim H. Nisancioglu, Michael A. Taylor, Tannecia S. Stephenson

**Affiliations:** 1 Department of Physics, The University of the West Indies, Kingston 07, JMAAW15 Jamaica; 2 NORCE Norwegian Research Center, and Bjerknes Center for Climate Research, 5008 Bergen, Norway; 3 Nansen Environmental and Remote Sensing Center, and Bjerknes Centre for Climate Research Bergen, 5006 Bergen, Norway; 4 Department of Earth Science, University of Bergen and Bjerknes Centre for Climate Research, 5020 Bergen, Norway

**Keywords:** Physical oceanography, Applied physics, Ocean sciences

## Abstract

Tropical Small Island Developing States (SIDS), such as those in the Caribbean, are among the most vulnerable to the impacts of climate change, most notably sea-level rise. The current sea-level rise in the Caribbean is 3.40 ± 0.3 mm/year (1993–2019), which is similar to the 3.25 ± 0.4 mm/year global mean sea-level (GMSL) rise (1993–2018). Throughout the year, Caribbean seasonal sea-level variability is found to respond to sea surface temperature variability. Over the past few decades, the trend in Caribbean Sea-level rise is also found to be variable. Satellite altimetry and steric sea-level records of the Caribbean region reveal a shift in the late 2003-early 2004, which separates two distinct periods of sea-level rise. Thermal expansion dominates the sea-level trend from 1993–2003. Following this period, there is an increased trend in sea-level rise, with a dominance of mass changes from 2004–2019, as confirmed by GRACE data. During this period, the sea-level trend is 6.15 ± 0.5 mm/year, which is 67% faster than the most recent estimates of global mean sea-level rise provided by the Intergovernmental Panel on Climate Change (3.69 ± 0.5 mm/year for the period 2006–2018). Despite its reduced importance, increasing temperatures contribute greatly to sea-level rise in the Caribbean region through thermal expansion of ocean water, hence there is a need to limit the current trend of global warming.

## Introduction

**Figure 1:**
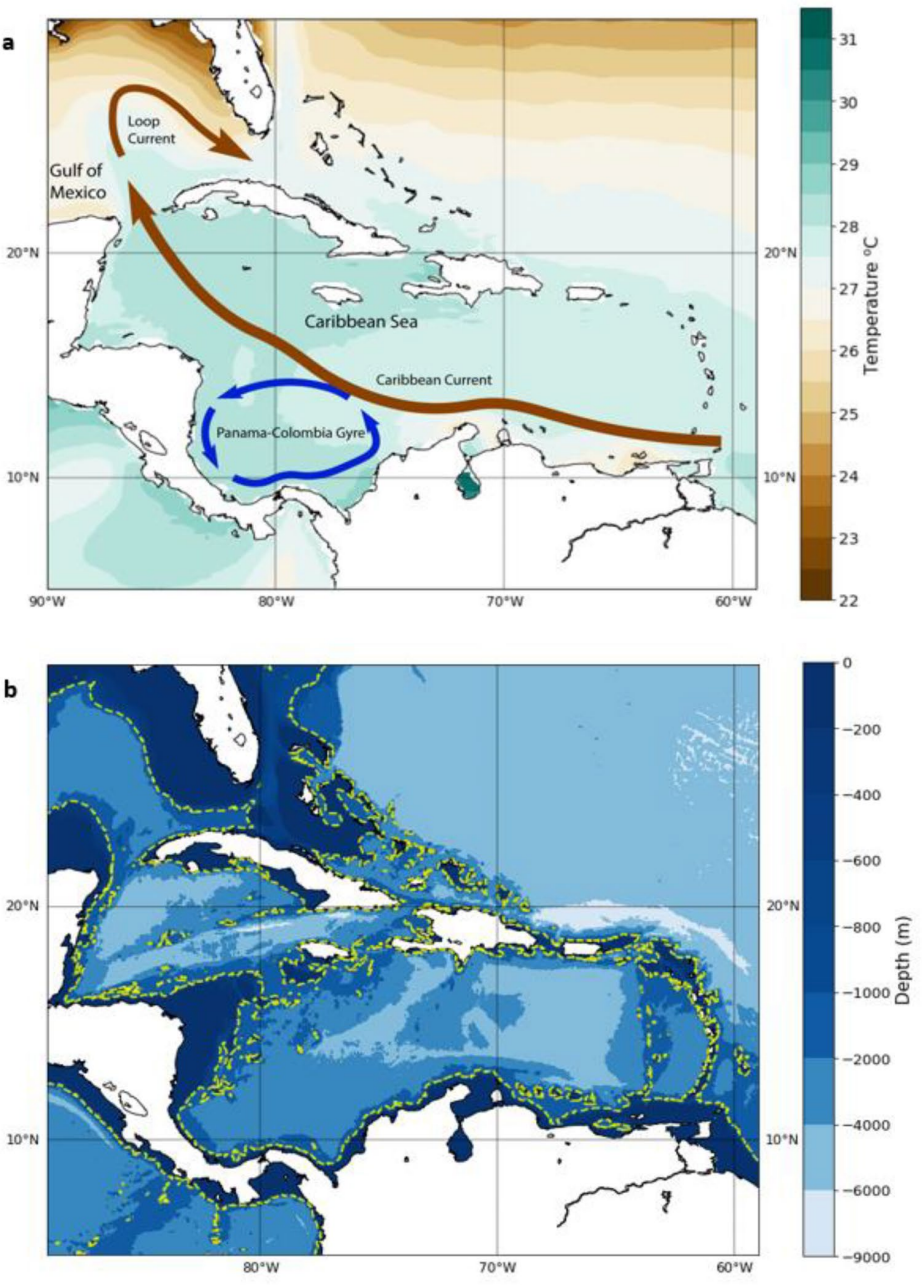
Caribbean Mean Sea Surface Temperatures (^o^C) from ERA5 for the altimetry era (1993–2019) and major current features. (**b**) Bathymetry of the Caribbean with 900m contour (‘--‘). (Created using: Python 3.9.12. URL: https://www.python.org/downloads/release/python-3912/).

Sea-level rise is one of the most severe impacts of climate change. The impacts are particularly dire for the Small Island Developing States (SIDS) in the tropics which are set to see significant destruction of coastal infrastructure and loss of coastal land within the twenty-first century^1^. The Caribbean region, with its relatively low elevation and dependence on the coastal zone for settlement, is extremely vulnerable to sea-level rise^2–4^. The Caribbean Sea, an eddy rich region^5–7^, is the largest marginal sea in the Atlantic Ocean with an average depth of 4400 m (Fig. 1b)^5,7^. A major circulation feature of the region, the Caribbean current, is essential to regional and the wider global climate system as it carries warm water from the Southern Atlantic inflow in the south-eastern Caribbean Sea and outflows in the Gulf of Mexico through the Yucatan strait^6^. This warm water is an important component of the Gulf Stream with impacts on the global climate system.

Sea-level rise in the Caribbean region has been found to be approximately 1.8 ± 0.1 mm/year for 1950–2009^8^ which is equivalent to the rise in global mean sea-level (GMSL) of 1.8 ± 0.5 mm/year from 1950–2009^9^. This relationship has been found to persist during the altimetry era, 1993- present where the recent trend in sea-level rise off the coast of Jamaica in the northern Caribbean (3.3 ± 0.4 mm/year), is slightly greater than the rise in GMSL (3.25 ± 0.4 mm/year)^10,11^. The major contributors to sea-level rise can be divided into two groups: steric (due to changes in density) and mass contributions also known as barystatic changes (due to melt of glaciers and ice sheets)^12^.. Cumulatively they are responsible for 89% of the sea level rise trends between 1993–2018 (Thermal expansion ~ 46%, Barystatic ~ 43%)^11^. The steric sea-level changes are caused by changes in temperature (thermosteric) and salinity (halosteric), which consequently translate to changes in sea-level. Sea-level change is not spatially uniform, and hence different regions experience different rates of sea-level rise. Spatial sea-level trend variability in the Caribbean is related to changes in regional ocean circulation systems such as the Panama-Colombia Gyre^13,14^ as well as with changes in the Caribbean Low-Level Jet (CLLJ) which may cause changes in wave height when it intensifies^15^. The signature of regional ocean circulation systems is also captured in the dynamic sea-level height, which is projected to change under different climate scenarios. Recent studies, based on resolution climate projections (e.g. Li et al.)^16^ show how changes in the Atlantic Meridional Overturning Circulation (AMOC) strength influence the North Brazilian and the Caribbean Currents, which in turn affect the dynamic sea-level field in the Caribbean Sea^17^.

The temporal variability in sea-level in the Caribbean has been found to be driven by steric changes and atmospheric (wind) forcing^10,18^. Thermal expansion has been found to be strongly correlated with ocean heat uptake, and thus increases in sea surface temperature^19^. The variability in Caribbean sea surface temperatures has also been found to be a key contributor to long-term variability in sea-level^10^. While interannually, Caribbean sea-level is observed to respond to large scale atmospheric oscillations such as the El Niño Southern Oscillation (ENSO)^18^ which modulates winds and the CLLJ in the Caribbean region^10,15^.

A change in the dominant forcing mechanism may invariably lead to different acceleration and deceleration periods in the rate of rise which have been identified in recent studies for different regions^20–22^. Notably, there is very little investigation of the acceleration of sea-level change for the Caribbean region, except for Ibrahim and Sun^6^, who investigated a 7 year period (2010–2016). There are also a few studies investigating the impact of steric sea-level change on Caribbean sea-level variability^8,14^. At the regional and local scale, steric sea-level rise is a crucial component of the overall sea-level change^23–27^. The thermosteric changes especially in the upper 900 m of the oceans play a significant role in sea-level rise on a regional scale^23–26^ and therefore this knowledge is essential to quantify regional sea-level change. Though smaller in amplitude, the halosteric contribution can still impact the trends, especially in semi-enclosed seas like the Caribbean Sea. Semi-enclosed basins such as the Mediterranean have shown greater influence from the halosteric component of steric-sea level rise^20,28^, hence it is also important to understand the halosteric influence in the Caribbean. In addition to steric influences, there may be different factors which contribute to temporal variability in sea-level on the regional and local scales^1,29^, including local atmospheric control, local ocean dynamic control and atmosphere–ocean interactions^11^.

This paper seeks to identify any anomalous shifts in the temporal variability of sea-level in the Caribbean and explore the impact of steric changes in the upper 900 m of the waters encompassing the Caribbean, on the region’s absolute sea-level change. We investigate the distinct periods of sea-level rise in the Caribbean and the difference in contributions of steric and barystatic sea-level change during these periods.

## Results

### Determining change point

**Figure 2:**
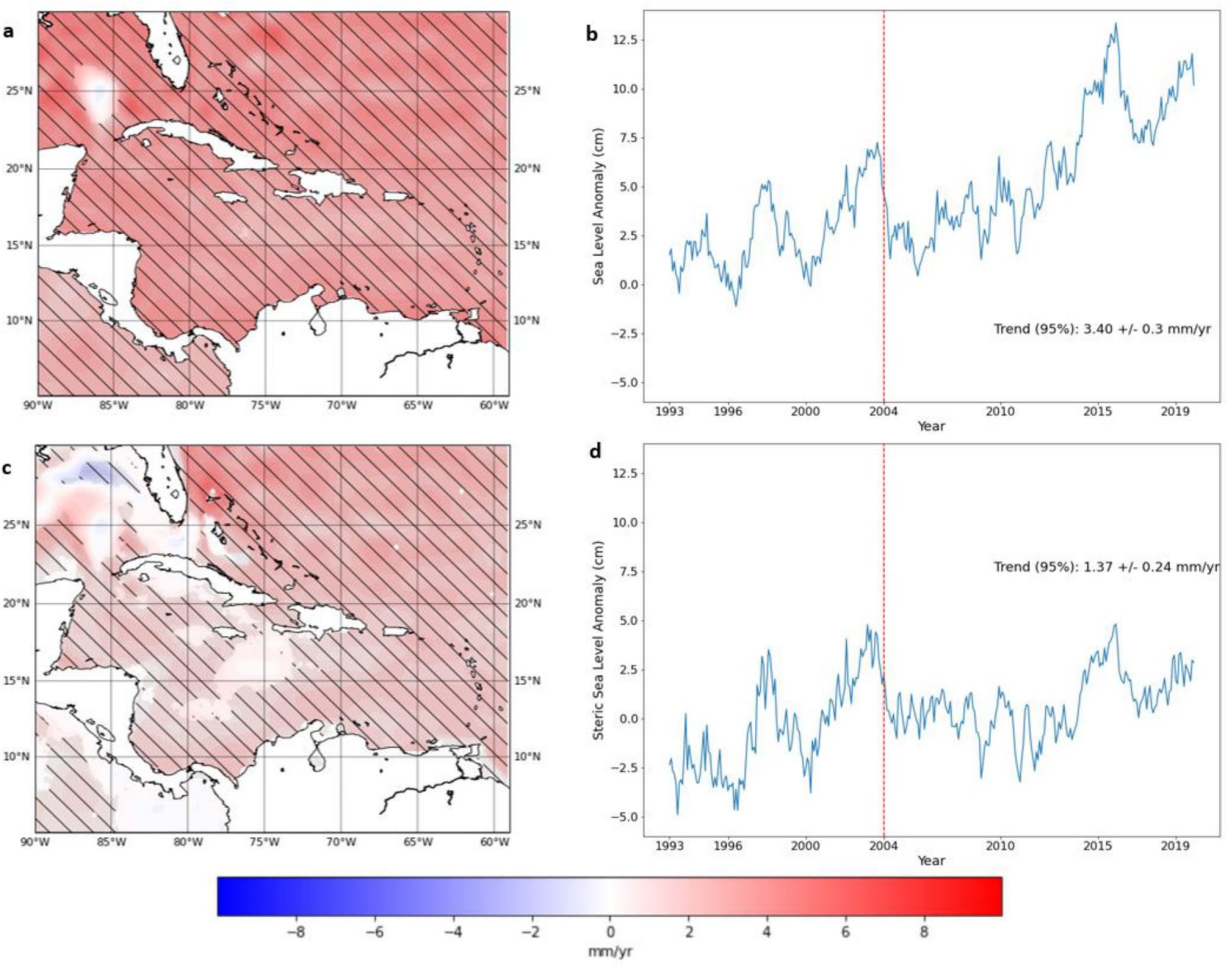
(**a**) Deseasoned absolute sea-level rise trends from altimetry for the extended Caribbean region; (**b**) Caribbean mean deseasoned sea-level anomalies timeseries; (**c**) Deseasoned Caribbean steric sea-level anomalies; (**d**) Caribbean mean deseasoned steric sea-level anomolies timeseries for the altimetry era (1993–2019). (Hatched areas represent statistically significant trends at 95% confidence level). (Created using: Python 3.9.12. URL: https://www.python.org/downloads/release/python-3912/).

Absolute sea-level rise from satellite altimetry in the extended Caribbean domain shown in Fig. 2a,c (hereafter refered to as *“Caribbean”*) is estimated at 3.40 ± 0.3 mm/year from 1993 to 2019. See ‘Methods‘ for details on defining the “Caribbean”. Figure 2a shows that during this period there is a general increasing trend in sea-level rise throughout the region, which agrees with earlier assessments of the sea-level change in the Caribbean region^10,14^. It also shows spatial variability in the rates of sea-level rise in the Caribbean. This variability ranges from a slight sea-level fall − 0.8 ± 3.5 mm/year. off the northwest coast of Cuba to over 6.0 ± 1.0 mm/year. over large portions of the region. This dipole at the entrance of the Gulf of Mexico may be related to the mesoscale variability of sea-level and Loop Current ring shedding in the Gulf. The water mass flowing north interacts with the steep topographic escarpments in the Gulf which may also introduce topographic steering^30^. The map of total steric trends shows spatial variability comparable to the trends for the absolute sea-level anomalies from altimetry (see Fig. 2c). Both timeseries of sea-level, averaged over the defined Caribbean region, show evidence of temporal variability in the record. There is an irregular shift in the sea-level in the months composing the end of 2003 and beginning of 2004 which coincides with the change point identified by Ibrahim and Sun^6^, Application of change point analysis through binary-segmentation^20^ confirms a common October 2003—March 2004 breakpoint across both times-series. The 2003–2004 change point may also be a part of the natural variability of sea level which has a strong influence on sea level in the Caribbean region through ENSO and the CLLJ as previously stated. The relatively short timeseries of 27 years used in the study means this possibility cannot be disregarded.

### Decomposing steric sea-level change

**Figure 3:**
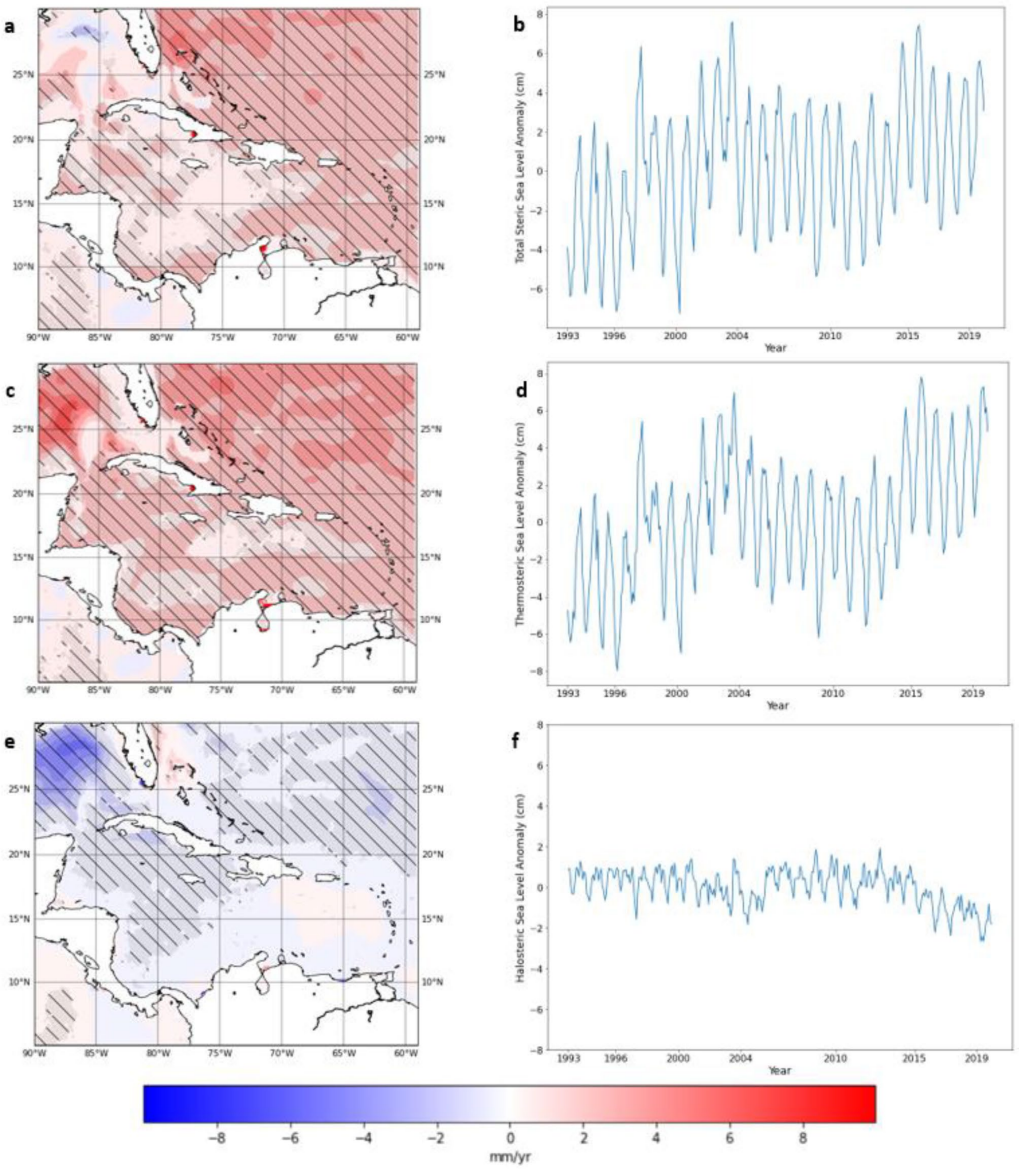
(**a**)Total steric trend map (**b**) Caribbean mean steric signal time series from 1993 to 2019, (**c**) Thermosteric sea-level trend map (**d**) Thermosteric sea-level time series (**e**) Halosteric sea-level trend map (**f**) Halosteric sea-level time series for Caribbean for the altimetry era (1993–2019). (Hatched areas represent statistically significant trends at 95% confidence level). (Created using: Python 3.9.12. URL: https://www.python.org/downloads/release/python-3912/).

Steric sea-level calculated over shallow continental shelves may be significantly smaller than over the deeper open ocean. This can be seen in Fig. 3c where the total steric trends are significantly smaller off the coast of Florida, Mexico and Nicaragua which coincide with the continental shelves in those areas (see Fig. 1b). The total steric sea-level trends in the Caribbean shows significant spatial variability (Fig. 3a). The rates of steric sea-level change in the Caribbean region range from − 2.4 ± 0.4 mm/year in the Gulf of Mexico to just above 5.0 ± 1.4 mm/year off the eastern coast of Florida. The regional mean rate of steric sea-level rise for the Caribbean is 1.37 ± 0.2 mm/year When the total steric sea-level is separated into the thermosteric and halosteric components, the dominant contributor is found to be the thermosteric component. The timeseries of the two components indicate that the thermosteric anomalies have an amplitude of 3–4 times that of the halosteric contribution (Fig. 3d,f). The trend map of the thermosteric shows a general positive trend throughout the region while the halosteric trend map shows a small negative trend regionally. There is a region of strong negative trend in the Gulf of Mexico seen in the halosteric trend map. This is largely counteracted by a large positive thermosteric trend. There is good agreement between the trends displayed on the map of thermosteric sea-level change and that of the absolute sea-level change from satellite altimetry (Fig. 2).

**Figure 4:**
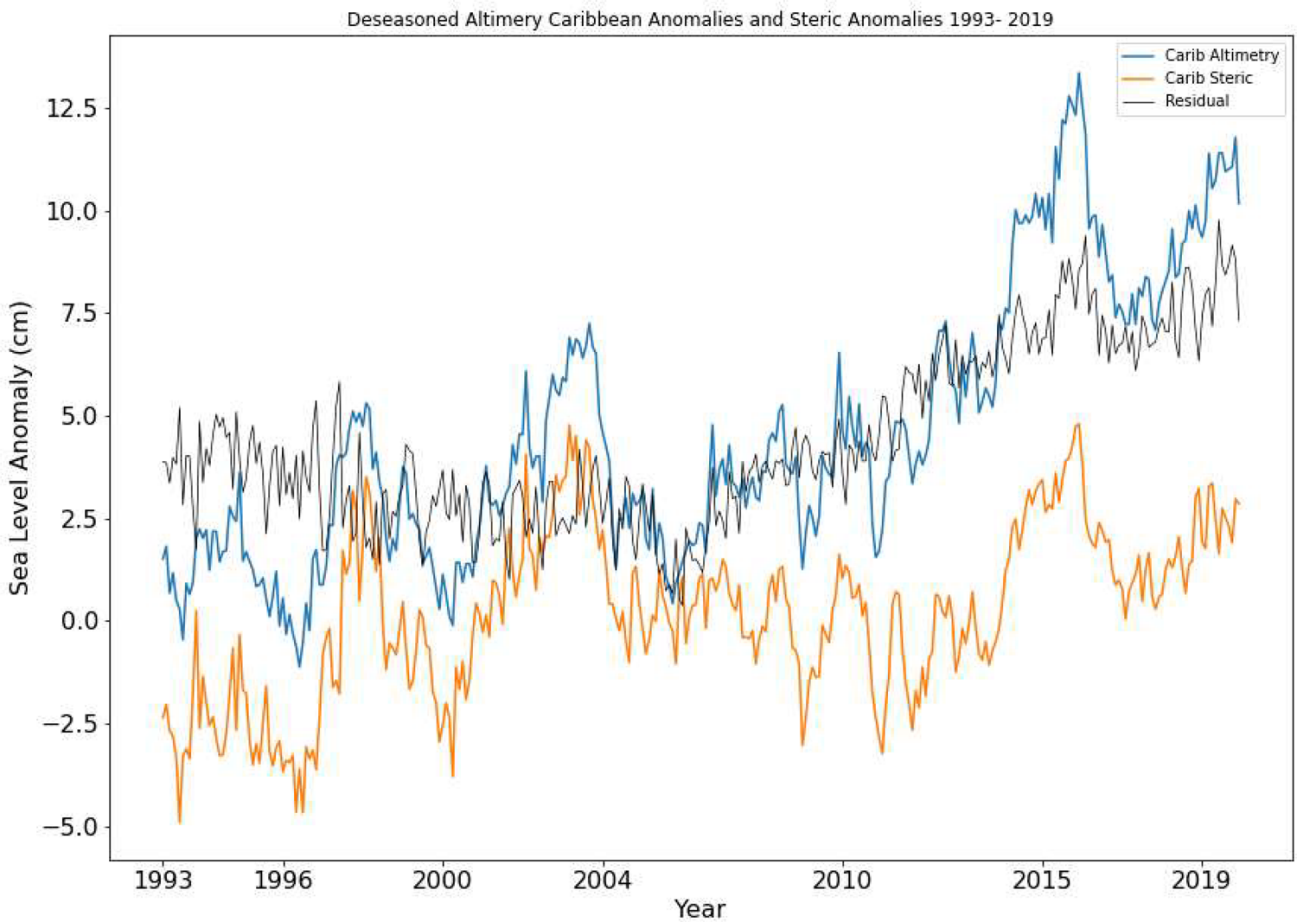
Deseasoned Caribbean absolute sea-level anomalies from altimetry, deseasoned steric anomalies and residual (Altimetry minus Steric). (Created using: Python 3.9.12. URL: https://www.python.org/downloads/release/python-3912/).

The timeseries of the total steric sea-level shows a clear seasonal signal (Fig. 3b). This is mirrored by the thermosteric component. Both showed largely increasing trends which were statistically significant at the 95% confidence level. The halosteric component is characterised by a declining trend for the last 10–15 years of the record. The halosteric map (Fig. 3e) showed that the trends were not statistically significant in the south and southeastern Caribbean. This may suggest the Caribbean Sea has experienced a trend of increasing salinity during this period. When the seasonal signal is removed from both the total steric and the absolute sea-level record from altimetry, a strong relationship between the two can be seen (Fig. 4). The correlation between the total steric sea level and the absolute sea level was found to be relatively large at 0.45 and statistically significant at the 95% confidence level.

**Figure 5:**
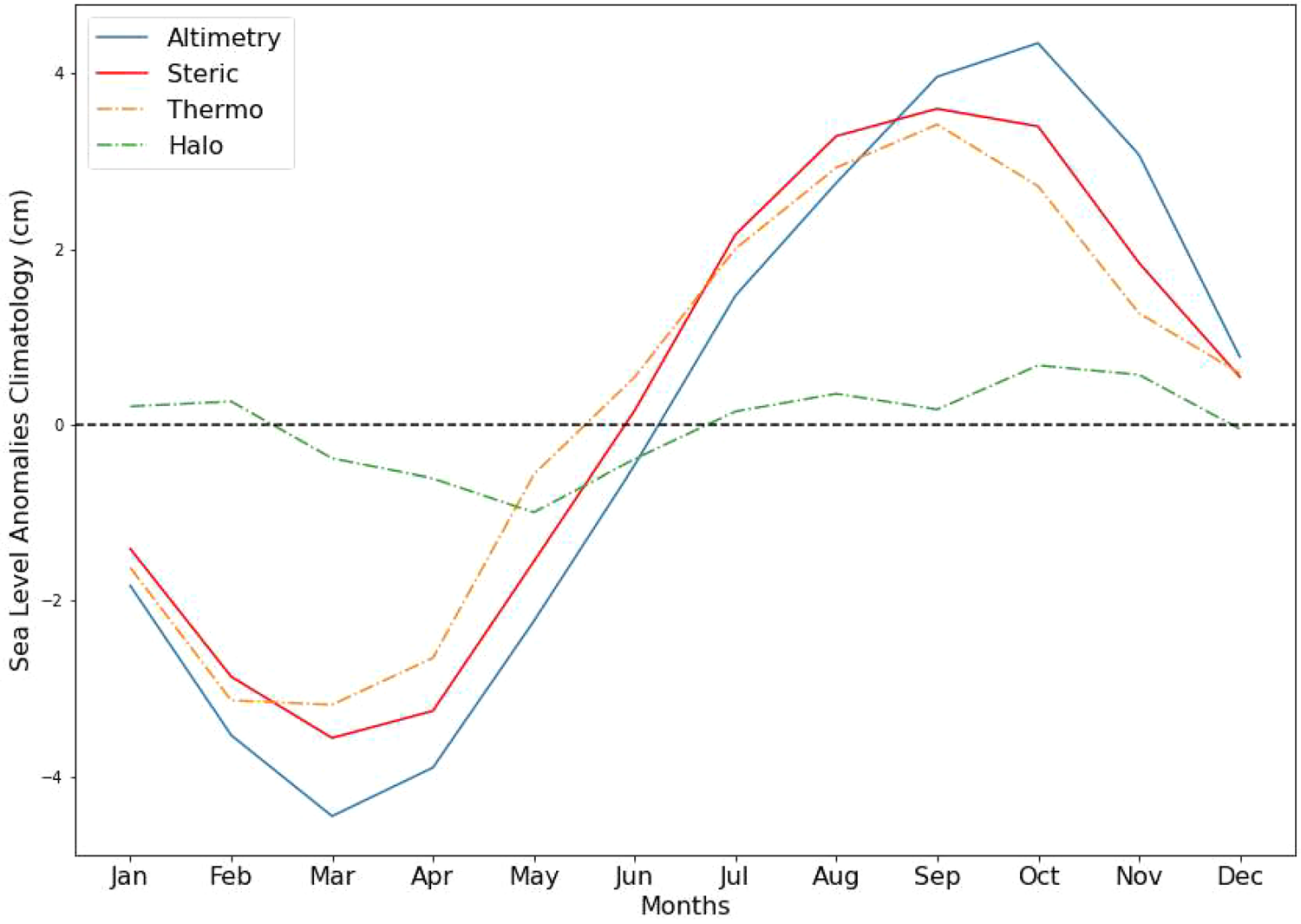
Climatology (seasonal/annual signal) of absolute sea-level anomalies from altimetry, total steric anomalies, thermosteric sea-level and halosteric sea-levels. (Created using: Python 3.9.12. URL: https://www.python.org/downloads/release/python-3912/).

Thermal expansion dominates the seasonal signal in the Caribbean, hence ocean temperatures play a crucial role in the increase in the sea-level rise in the region. The climatology (seasonal variability) of the absolute Caribbean sea-level (see Fig. 5) shows a pattern of a trough in early northern hemisphere spring (March). The temperatures build to a single peak in September, and decrease as winter approaches. This seasonal variability is also seen in the steric sea-level change and the sea-level anomalies (see Fig. 5). The steric sea level peaks in September, while the absolute sea level peaks a month later. Both the total sea-level anomalies and the steric sea-level anomalies are at a minimum in March, as opposed to the February minimum seen in Caribbean ocean temperatures. The general agreement between the three is further evidence of a strong relationship between the ocean temperatures, steric sea-level change and absolute sea-level variability in the Caribbean as was suggested by previous studies^10,13^.

### Caribbean sea-level rise before and after the 2003–2004 shift

The 2003–2004 shift was identified in the sea-level anomalies from altimetry and steric time series. This section will examine 2 time periods 1993–2003 and 2004–2019. Table 1 shows the linear trends in sea-level change for 1993–2003, 2004–2019 and the overall study period. It is followed by the evaluation of the differences in steric sea-level change and its relation to the sea-level variability during 1993–2003 and 2004–2019. The altimetry trends have been adjusted for glacial isostatic adjustment (GIA)^31^.

**Table 1:** Caribbean mean linear trends in sea-level change from satellite altimetry (C3S), total steric sea-level rise, halosteric Sea-level change and thermosteric sea-level change (ARMOR3D).

Trends (mm/yr)	1993–2019	1993–2003	2004–2019
Altimetry	3.40 ± 0.3	4.08 ± 0.8	6.15 ± 0.5
Steric	1.37 ± 0.2	5.58 ± 0.9	1.59 ± 0.4
Halosteric	− 0.49 ± 0.08	− 0.51 ± 0.2	− 1.03 ± 0.2
Thermosteric	1.86 ± 0.3	6s.08 ± 0.9	2.61 ± 0.5

#### 1993–2003

**Figure 6:**
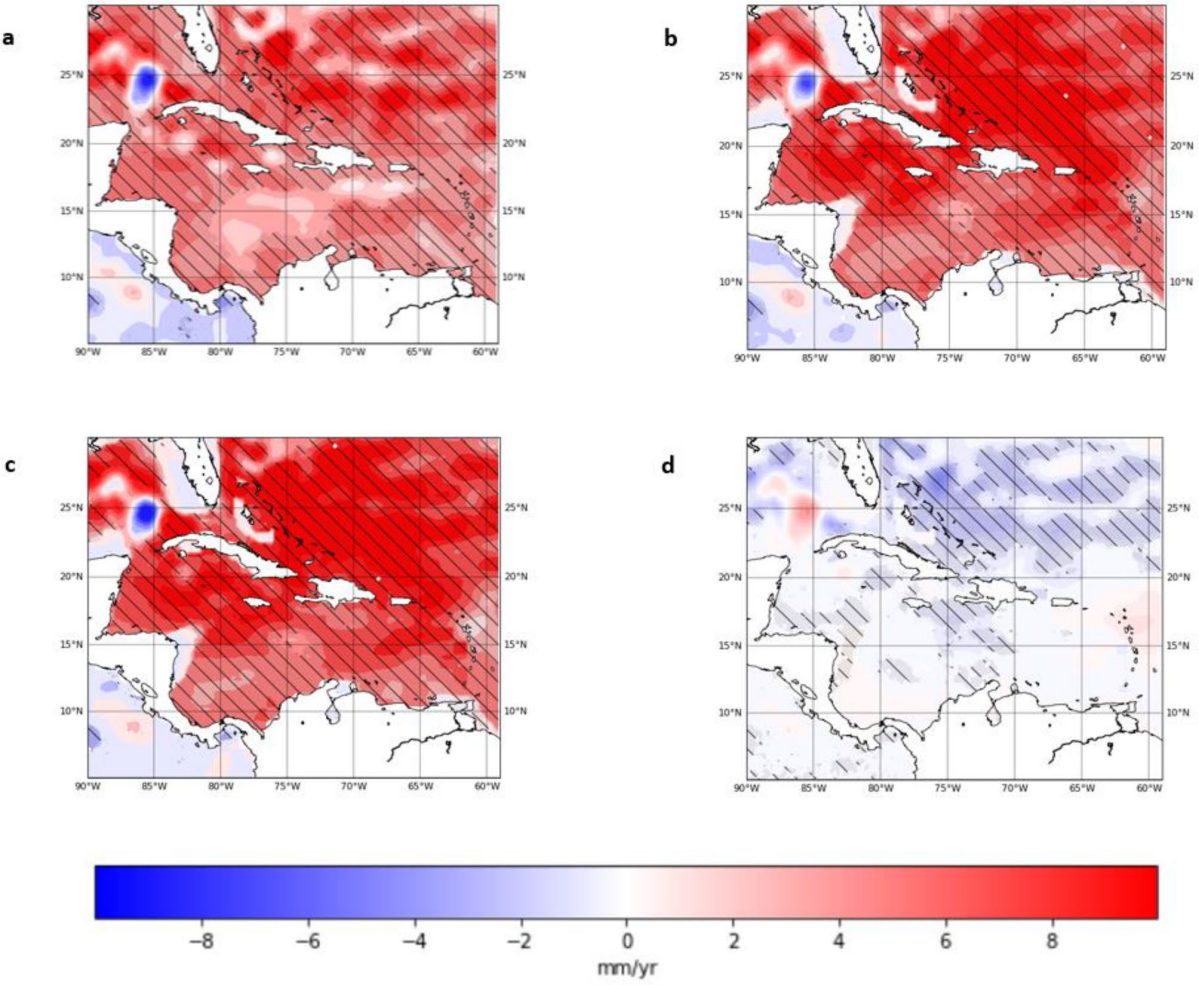
(**a**) Absolute sea-level anomalies trend (**b**) Steric sea-level anomalies trend (**c**) Thermosteric sea-level anomalies trend (**d**) Halosteric sea-level anomalies trend from 1993–2003. (Hatched areas represent statistically significant trends at 95% confidence level). (Created using: Python 3.9.12. URL: https://www.python.org/downloads/release/python-3912/).

Figure 6 shows the trend in the altimetry data for 1993–2003. There is a broad agreement between absolute sea-level and steric sea-level trend maps during 1993–2003 (Fig. 6a,b). The maps show a strong positive overall trend in sea-level during 1993–2003 in both records (see Table 1). The steric sea-level rise trend component is larger than that of the absolute sea-level rise trend. The steric trends are however distinctively smaller but still positive on the continental shelf areas off the coast of Florida, Mexico, and Nicaragua (Fig. 6b,c). The maps also show consistent spatial variability with a distinct dipole in the Gulf of Mexico and a region of high sea-level trends just north of the Bahamas, seen in both trend maps. Within the northern Caribbean, the steric sea-level trends are higher than absolute trends by 2–3 mm/year The thermosteric sea-level change is spatially more variable than the halosteric. The thermosteric sea level trends were also statistically significant for the majority of the Caribbean however only the northeastern section of the domain showed statistically significant halosteric trends. There is a significantly larger range in the thermosteric than the range of the halosteric sea-level change, which lies between ± 5 mm/year. This is reflected in the mean Caribbean trends, where the thermosteric trend was found to be 6.08 ± 0.9 mm/year while that of the halosteric was found to be -0.51 ± 0.2 mm/year.

#### *2004*–*2019*

**Figure 7:**
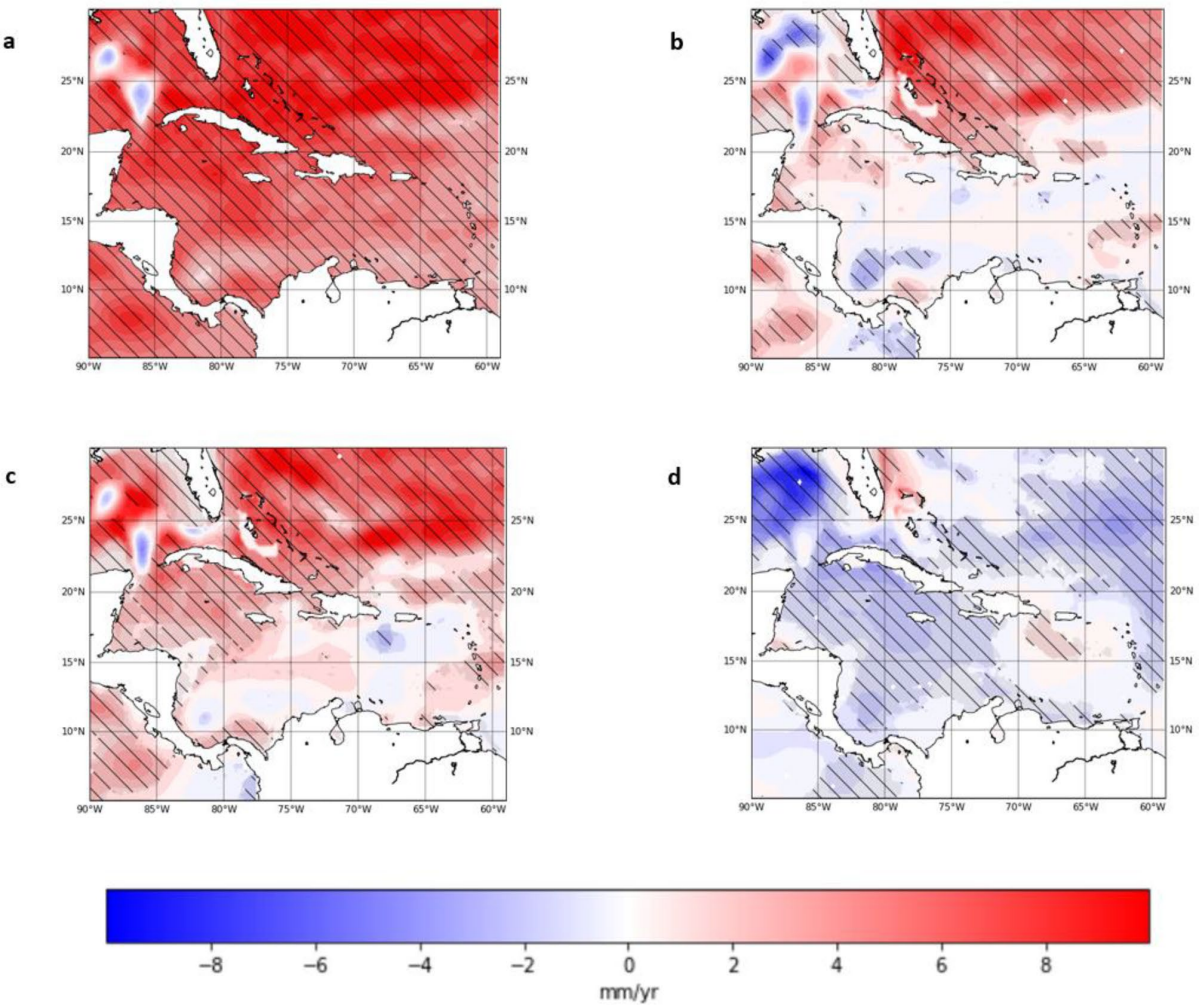
(**a**) Absolute sea-level anomalies trend (**b**) Steric sea-level anomalies trend (**c**) Thermosteric sea-level anomalies trend (**d**) Halosteric sea-level anomalies trend from 2004 to 2019. (Hatched areas represent statistically significant trends at 95% confidence level). (Created using: Python 3.9.12. URL: https://www.python.org/downloads/release/python-3912/).

For 2004–2019, the mean Caribbean absolute sea-level rise trend from altimetry is 6.15 ± 0.5 mm/year when adjusted for GIA while the mean Caribbean steric sea-level rise trend is 1.59 ± 0.4 mm/year. This is equivalent to an acceleration in altimetry of 0.1 mm/year^2^ in the absolute sea-level while the steric sea-level rise saw a deceleration of − 0.27 mm/year^2^. The total steric and thermosteric trend maps, that show that most of the Caribbean Sea has seen insignificant steric trends below 5 mm/year. (Fig. 7b,c). The northeastern section of the Caribbean domain was a notable exception which showed significant increasing trends in steric sea level for 2004–2019. This region also shows the highest positive trends in sea-level rise on the map of trends from altimetry (Fig. 7a). As opposed to the previous period, the halosteric sea level trends were significant in most of the Caribbean except for the southeastern and northcentral Caribbean. The differences between trends in 2004–2019 and 1993–2003 may suggest a reduction in the contribution of the total steric sea-level change to the absolute sea-level rise in the Caribbean in the later years of the record. This is further elaborated on in the discussion.

Thermosteric sea level shows a reduction in the mean Caribbean trend to 2.61 ± 0.5 mm/year. whereas halosteric sea level shows a two-fold increase in the Caribbean mean to − 1.03 ± 0.2 mm/year. Consequently, the overall reduction in the total steric contribution to Caribbean mean sea-level change is a result of decreased thermosteric contribution which causes smaller positive trends in the region and increased halosteric contribution which exhibit largely negative trends in the Caribbean. There was however a continued trend of increasing temperatures in the Caribbean region in 2004–2019.

**Figure 8:**
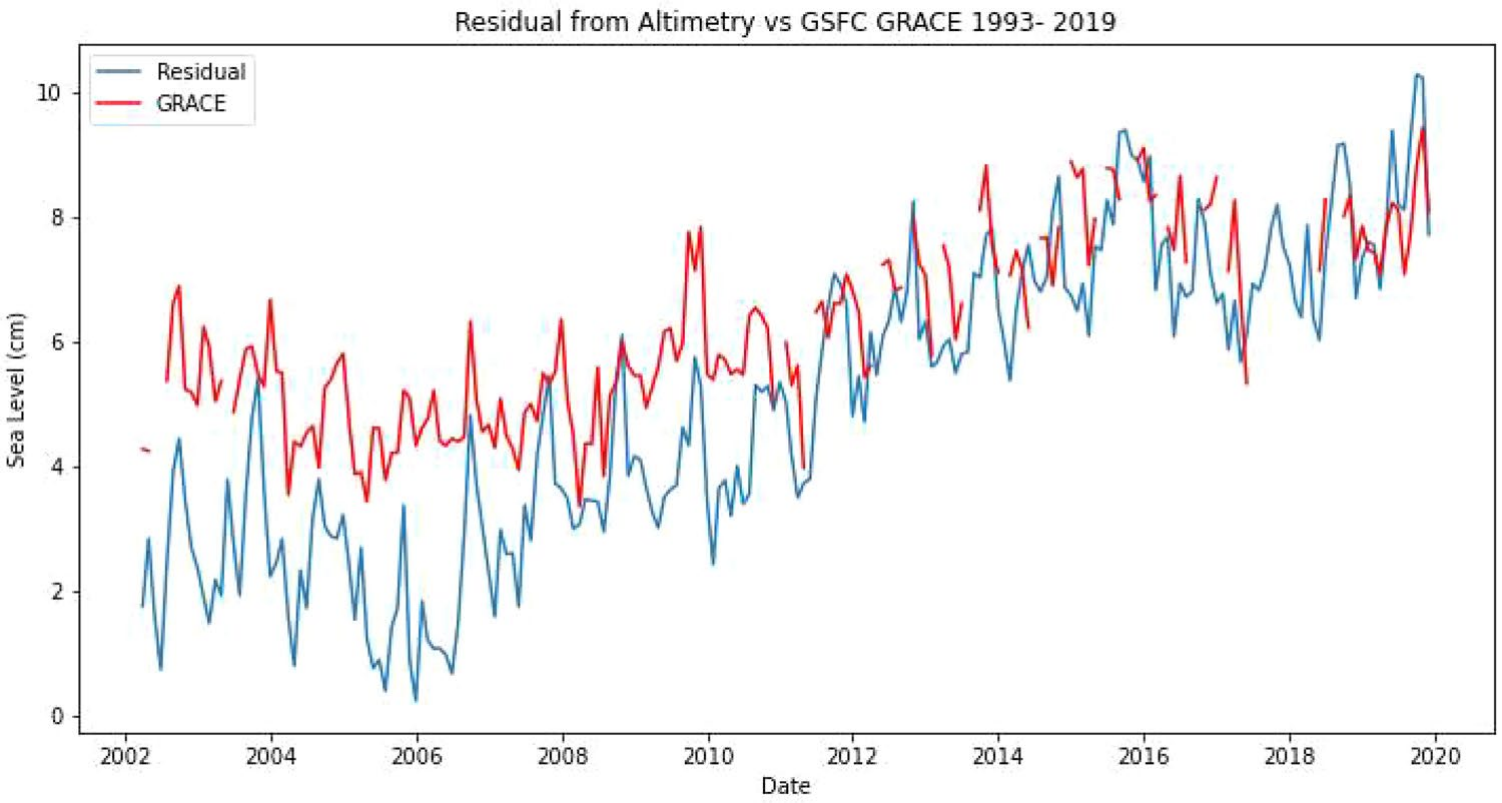
Residual timeseries (Absolute SL minus Steric SL) and Mass equivalent change in sea-level from GRACE (GFSC Grace). (Created using: Python 3.9.12. URL: https://www.python.org/downloads/release/python-3912/).

Though this paper is not focused on a sea-level budget analysis for the region, the difference between the absolute sea-level change and the steric component (i.e. the residual) would refer to the mass component of sea-level rise (Fig. 4). This shows a strong upward trend in the later part of the focus period. There is an increasing trend in both the residual signal (absolute sea level minus Steric sea level) and the mass equivalent sea-level change, which is clearly identifiable during 2004–2019. Figure 8 shows the comparison between the residual trends and observed mass change data from GRACE from 2002 to 2019. To account for the differences in lifetimes and baseline references between the Altimetry and GRACE missions, the temporal mean of the residual signal for 2002–2019 is added to the GRACE estimates. For the 7 year period of 2004–2010, the longest continuous period of GRACE data, the trend in the absolute sea-level rise was 4.3 ± 0.4 mm/year while the GRACE mass equivalent trend was 2.3 ± 0.8 mm/year. After removing the linear trend and seasonal signal a positive correlation of 0.18 was found between the two variables this period. This correlation was statistically significant at 95% confidence level. For the overall record between 2002–2019 a correlation of 0.26 (also significant at a 95% confidence level) was found between the absolute sea-level change residual and the mass change component of the sea-level change when missing values are ignored. This will be briefly expounded upon in the discussion section.

## Discussion

Long-term sea-level rise can be detected at the global scale but regional drivers (e.g. temperature and salinity, ocean circulation, wind) can lead to departures from the global sea-level trend and influence the sea-level variability at the seasonal and inter-annual temporal scales. Barystatic sea-level rise (sea-level changes due to changes in ocean mass) and steric changes are the main causes of the increasing sea-level rise globally. Regionally, their relative contribution to the absolute sea-level change may vary. Hence, identifying the impact of rising temperatures on the spatial and temporal variability of regional sea levels requires deeper scrutiny.

Regionally variability in rates of sea-level rise have become an area of focus for recent studies^20–22^ in part due to the advent of technology such as satellite altimetry, ARGO profile floats and GRACE + GRACE-FO. This has allowed us to investigate the major components of global mean sea-level individually and collectively as a sea-level budget. Thermal expansion explained approximately 40%-50% of the global sea-level rise between 1971 and 2018^12,39,40^. For the period of 1993–2019 the overall steric trend for the Caribbean region is found to be 1.37 ± 0.2 mm/year. This is equivalent to about 40% of the overall trend found in the altimetry record for the region. The contribution of the thermosteric and halosteric components of the steric sea-level changes to the overall steric response may differ for different regions^12,20^. In the Caribbean region there is a long-term increasing trend in sea-level^10,14^., however the changes are found to vary temporally on seasonal and interannual scales. This study shows that this seasonal and interannual variability in absolute sea-level is largely driven by the thermosteric component, while the mass component modulates decadal variability and most likely the non-linear trends. The results show that the dominant contributor to steric sea-level rise is the thermosteric sea-level component (Fig. 5). The magnitude of the thermosteric contribution to the total steric sea-level change was three to four times the contribution of the halosteric contribution throughout the entire study period. Thermosteric sea-level trends were reduced by more than 50% to 2.61 ± 0.5 mm/year during 2004–2019, thus reducing the overall steric sea-level contribution to absolute sea-level rise trends during 2004–2019 which will be discussed further below.

While halosteric sea-level change is negligible on the global scale it may be important regionally^12^. In the Caribbean, halosteric sea-level changes are not negligible (see Fig. 5) and can partly offset thermosteric trends. Evaporation in the Caribbean typically exceeds the precipitation, thus making the region a net evaporation basin^6,41^. In the long term, this translates to the water within the Caribbean region becoming more saline^11^. This is also reflected in the downward trend in the halosteric contribution (Fig. 3f and Fig. 7d) during 2004–2019 due to the resulting haline contraction. On shorter time scales, there is much variability in the halosteric contribution (Fig. 3f). Interannual variability could be attributed to variations in salinity in the upper layers of the Caribbean Sea due to the inflow of fresher water from the Atlantic. This is fuelled by pulses of fresh water from the Amazon and Orinoco Rivers which are transported north by the North Brazil Current (NBC) and the Guyana Current (GC) then advected westward through the Caribbean region^41,42^. This freshwater mixes with the saltier water of the Caribbean Sea and causes variability in surface salinity. This surface salinity anomaly may then be vertically mixed with lower levels in the water column below the surface^6^. The seasonal signal seen in this salinity time series may be attributed to the seasonal freshwater discharge from the Magdalena (Colombia) and Atrato (Colombia) which together provide 60% of all the water discharged into the Colombia Basin. The Colombia Basin forms a large portion of the wider Caribbean Basin^41,43^. The magnitude of the overall halosteric contribution is not enough to drive the seasonal changes in sea-level but is currently too large to be negligible. With climate change, the trend of increasing salinity seen in 2004–2019 (Fig. 3f and Fig. 7d) is projected to continue in the twenty-first century as the waters of the region tend to be more saline^11^. Therefore, the halosteric sea-level changes may increase in relevance and importance in the coming decades. On the interannual scale, local winds and the impact of Caribbean Low Level Jet (CLLJ) also have a significant impact on sea-levels in the Caribbean region. ENSO modulates the strength of both the trade winds across the region and CLLJ with the warm (cool) phase causing a shift in the trade winds as well as a strengthening (weakening) of the CLLLJ which results in increases (decreases) in sea-levels in the Caribbean^10,44^. The CLLJ can enhance upwelling with stronger winds during ENSO warm phase events^41,45^. This may increase the cold saline water in the Caribbean region thereby influence the mixed layer depth and the heat budget in the Caribbean region^46^. In this way ENSO also indirectly influences the halosteric contributions. With continued climate change, the projected decrease in AMOC strength in the coming decades may also impact Caribbean sea levels through the reduction of the eddies propagating into the region after breaking off the North Brazil Current^17^. This may reduce the dynamic sea-level extremes and result in slightly lower sea levels in the region.

**Figure 9:**
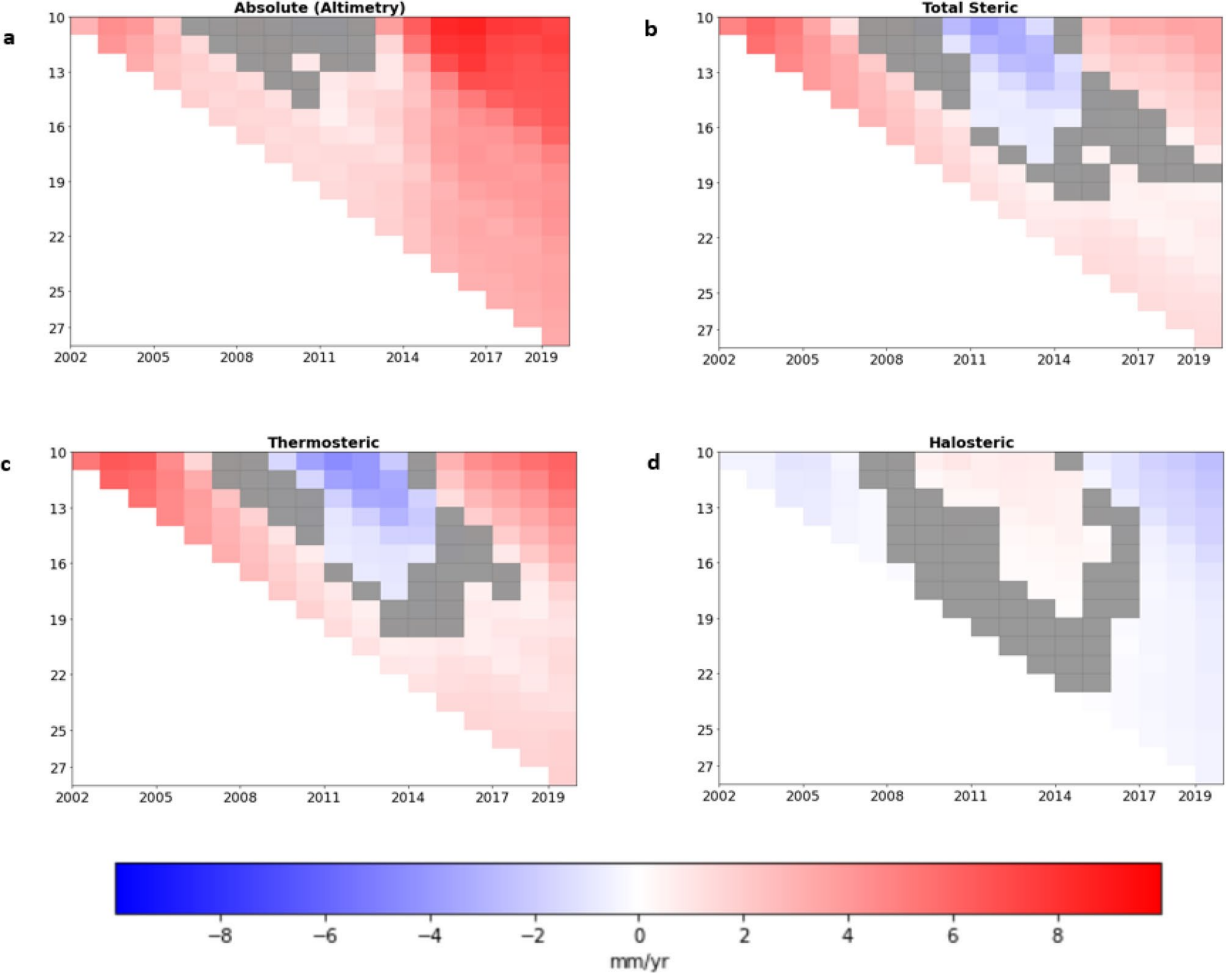
Sensitivity Analysis of Caribbean mean (**a**) Absolute (Altimetry) (b) Total Steric sea level trends (**c**) Thermosteric sea level trends (**d**) Halosteric sea level trends from 1993 to 2019. The y-axis represents the number of years used to calculate the trend while the x-axis represents the year at which the trend calculation ends. At the change point 2003- 2004 (y = 11 and x = 2003), there is a 11 year trend calculated from the start of the date in January 1993 to December 2003. The grey regions represent trends that are not statistically significant at the 95% confidence level. (Created using: Python 3.9.12. URL: https://www.python.org/downloads/release/python-3912/).

When the strong seasonal cycle of the sea-level is removed from the satellite altimetry record for the Caribbean change point analysis using binary-segmentation^20,47,48^ (See Methods) reveals a changepoint centred around 2003–2004 (Fig. 2c). The shift identified at this time coincided with a downward shift in the daily miniumum temperatures in parts of the Caribbean basin^49^ and a cooling period in the SSTs in the southern Caribbean basin^50^. These changes in temperature and salinity may be responsible for the shift due to the strong impact of steric influences in the Caribbean region. However the region experiences strong natural variability of sea levels and thus more research is needed to reach any definitive statements on causation or attribution. Sensitivity analysis of the trends (Fig. 9) show that trends calculated for the two periods vary with the position of the change point however the major components of absolute sea-level were distinct in 1993–2003 and 2004–2019. In 1993–2003, the total steric trends are slightly greater than the sea-level rise trends shown in the absolute sea-level (Table 1, Fig. 6a,b). The overall magnitude of the steric component is within 2-3 cm of the absolute sea-level variability during this period (Fig. 4) which suggests that the steric component was responsible for a significant portion of the variability. The temperature in 1993–2003 is slightly cooler than the overall mean temperature of the region for 1993–2019, however the trends in thermosteric sea-level rise are greater in 1993–2003 at 6.08 ± 0.9 mm/year than in 2004–2019 at 2.61 ± 0.5 mm/year. This trend is also seen globally as global sea surface temperature continue to rise however the thermosteric contribution forms a smaller portion of the recent global mean sea-level rise^11^. With the increase in the length of satellite altimetry data there has been more robust analysis which has also confirmed the presence of this trend of accelerating SLR^51–54^. The period following the 2004 change point, 2004–2019, is characterised by a 0.1 mm/year^2^ acceleration in the absolute sea-level rise for the Caribbean region. This is in line with previous estimates found by Hamlington et al.^55^, for 2006–2018. The Caribbean mean sea-level trend for 2004–2019 was 6.15 ± 0.5 mm/year, which is 67% faster than the estimate of GMSL rise of 3.69 ± 0.5 mm/year for the same period^11^.

Our results show that for both 1993–2003 and 2004–2019 the rate of sea-level rise in the Caribbean exceeded these estimates. The acceleration in sea-level rise in 2004–2019 can be largely attributed to total mass loss of land ice which is the highest contributors to GMSL during this time^11,56^. This increase in contribution of ice sheets and glaciers is the biggest factor in divergence between the steric and the altimetry sea-level anomalies seen in the 2004–2019 (see Fig. 5). The deceleration of − 0.27 mm/year^2^ in the thermosteric sea-level rise trends during 2004–2019 indicates this slower increase which agrees with the finding of AR6^11^. While thermosteric contribution continues to increase, it is at a slower rate than that of the absolute sea-level change^57,58^. However, the 2.61 ± 0.5 mm/year. trend of thermosteric sea-level rise in the Caribbean in 2004–2019 is 2 times the rate of global thermal expansion between 2006 and 2018 which was estimated at 1.39 ± 0.7 mm/year^11^.

We found a significant upward trend in the residual signal (Absolute minus Steric) for the Caribbean region, during 2004–2019 (Fig. 8). This points to an increase in the mass components contribution to sea level rise in the Caribbean. Trend analysis between the residual signal and GRACE data revealed a similar increasing upward trend in the mass component but smaller than the residual. After removing the linear trend and seasonal signal a positive correlation of 0.26 was found between the residual and mass component for the entirely of the GRACE era (2002–2019). The analysis confirms the increasing trend in mass contribution to the sea-level rise in the Caribbean during 2004–2019. The difference between magnitude and trends in the mass equivalent sea-level change component and the residual suggests that there may be other local factors which may be responsible for the additional increase in sea-level rise. In addition to the vast topographical and bathymetric differences withing the Caribbean (Fig. 1b), 1993–2003 also precedes the era of GRACE. The topographical setting may influence sea-level change and therefore caution is advised when making inferences of the mass contribution to the absolute sea-level change. Quantifying the contribution of other local factors like wind, vertical land movement, glacial isostatic adjustment and others may indicate if the Caribbean SLB can be closed within uncertainties^57,59^. That is the logical next step in future work however it is beyond the scope of this paper.

## Conclusion

The Caribbean region is a perfect example of the inhomogeneous and dynamic nature of sea-level change. The region experiences seasonal, interannual and long-term variability in sea-level over the past few decades. An irregular shift in temporal variability is identified in the region and centred in late 2003 to early 2004. This divides the altimetry record into an early period, 1993–2003 and a late period 2004–2019. The Caribbean mean sea-level rise from 1993 to 2019 was found to be 3.40 ± 0.3 mm/year while the rate of sea-level rise during 2004–2019 was 6.15 ± 0.5 mm/year, which is 67% faster than the GMSL rise (3.69 ± 0.5 mm/year.) for the same period.

Thermosteric sea-level change is the major driver of seasonal sea-level variability in the Caribbean throughout the record and has a magnitude that is 3–4 times the magnitude of the Halosteric contribution to the total steric sea-level rise. Steric sea-level change is the major contributor to Caribbean absolute sea-level rise during 1993–2003 , however in 2004–2019 the relative importance of thermosteric sea-level rise decreases. Instead, the mass change contribution to Caribbean absolute sea-level rise is found to be the dominant component after 2004. This is attributed to an increase in the mass equivalent sea-level due to loss of land ice from glaciers and the ice-sheets on Greenland and Antarctica.

The Caribbean is mainly composed of Small Island Developing States (SIDS) which are moderately to extremely vulnerable to sea-level rise. The significantly higher rate of sea-level rise in the Caribbean region is of special concern because of the high concentration of population, infrastructure, and economic resources located in the coastal zone of Caribbean Islands^60–63^. This faster rate of sea-level rise could mean greater destruction of coastal infrastructure most notable tourism infrastructure on which many Caribbean islands are highly dependent for income^63^. On the local and regional scale there remains a dependence on tide gauge data to do analysis of local relative sea-level changes. The Caribbean region is largely lacking in long, high quality tide gauge data however satellite altimetry can be used a reliable representation of the absolute sea-level change in Caribbean region^10,64,65^ and is used in this study. Efforts to remedy this data gap continue in the region and with this in mind future work may include robust break point analysis and further examination of the results of this paper^66^ using time series of local tide gauge data, when they reach the length and quality thresholds. This study calls attention to the rapidly changing characteristics of Caribbean sea-level rise while setting the stage for a future, in depth look at the possibility of a sea-level budget closure for the Caribbean, within uncertainties.

### Data

#### Satellite altimetry

The satellite altimetry dataset used in this paper was provided by Copernicus Climate Change Service (C3S). This is the 0.25°*0.25° gridded dataset of monthly sea-level anomalies from 1993 to 2019 which is optimally interpolated so that it is stable and ideal for climate change analysis^20,32^. The data was processed and corrected for various atmospheric and instrumental biases including sea state bias and instrumental drift among others. This dataset has been found to be appropriate for application in assessing long term changes in the ocean relating to climate change and has been used in regions lacking long tide gauge records such as the Caribbean^10,20^. For further details on processing and corrections please see^32^. The sea-level trend maps and timeseries are not adjusted for vertical land motion and glacial isostatic adjustment (GIA) due to the heterogeneous nature of techtonic movement on the Caribbean plate^6,67^ which may have different relative sea-level rise impacts at different points in the region. The Caribbean mean value for GIA, calculated using the ICE-5G(VM2)^31^, was 0.07 mm/year. This has been added to the altimetry trend values in Table 1, to account for GIA in the mean sea level trends for the region.

#### Steric sea-level

Steric sea-level contribution to sea-level change was calculated using the temperature and salinity profiles provided by ARMOR 3D reprocessed multivariate ocean state estimate dataset. ARMOR 3D is a gridded dataset with monthly temporal resolution and a 0.25°*0.25° spatial resolution of temperature and salinity data. It uses both satellite and in situ observation to produce an optimal interpolated data set of ocean temperature and salinity at 49 non-equidistant depth levels down to 5500 m^68–70^. It reconstructs these profiles using a three-step process which begins with sea-level anomalies and sea surface temperatures from satellites projected downwards to create vertical profiles using a multiple linear regression method. In situ observations of T/S vertical profiles (including Argo, CTDs, mooring measurements etc.) are then combined with these generated profiles through an optimal interpolation method^70,71^. The ARMOR3D dataset was used previous by Ibrahim and Sun^6^ to investigate the acceleration in sea-level rise in the Caribbean in the 2010s. The World Ocean Atlas (WOA18) has also been used in previous studies of the Caribbean region to reexamine the climatology and water masses the Caribbean Sea^34^. WOA18 climatology is incorporated into the ARMOR 3D data as a means of calibration which serves as further validation for its use in the Caribbean region.

#### GRACE data

The Gravity Recovery and Climate Experiment (GRACE) dataset used in this study was provided by NASA Goddard Space Flight Center (GSFC). The data supplied is mascon solutions of 1-arc-degree equal area cells which have been corrected for glacial isostatic adjustment (GIA), tides (solid earth and ocean) and other standard atmospheric and ocean corrections. For more details on dataset and corrections see^37^.

#### Additional data

Table 2 shows further information on all the datasets used for the study including sea surface temperature estimates.

**Table ﻿2:** Details of Satellite Altimetry (CMEMS), Bathymetry, Sea Surface Temperature, Ocean Salinity, Ocean Temperature and Gravity Recovery and Climate Experiment (GRACE) datasets used in the study.

Data Type	Details	Length	Source	Reference
Satellite Altimetry	Daily Data Sea surface height above mean Sea Surface (‘sla’ sea-level anomaly). 0.25°*0.25° Gridded Dataset	1993–2019	Copernicus Climate Change Service (C3S): https://cds.climate.copernicus.eu/cdsapp#!/dataset/satellite-sea-level-global?tab=overview	^32,33^
Sea Surface Temperature	European Centre for Medium-Range Weather Forecasts(ECMWF), ERA5 reanalysis of temperature of sea water near surface Monthly 0.25° × 0.25° Gridded Dataset	1993–2019	https://cds.climate.copernicus.eu/cdsapp#!/dataset/reanalysis-era5-single-levels-monthly-means?tab=overview	^22^
Salinity and Temperature	World Ocean Atlas Climatology (WOA18) derived from objectively analysed and quality-controlled observations	1995–2004	https://www.ncei.noaa.gov/access/world-ocean-atlas-2018/	^34,35^
Salinity and Temperature	ARMOR 3D Reprocessed dataset of interpolated in-situ and satellite observed salinity and temperature data 0.25°*0.25° Gridded Dataset	1993–2019	https://data.marine.copernicus.eu/product/MULTIOBS_GLO_PHY_TSUV_3D_MYNRT_015_012/description	^6,36^
GRACE	Gravity Recovery and Climate Experiment (GRACE, GRACE-FO). Mascon solutions of 1-arc-degree equal area cells	2002–2019	GFSC Laboratory (NASA) -https://earth.gsfc.nasa.gov/geo/data/grace-mascons	^37^
Bathymetry	General Bathemetric Chart of the Ocean (GEBCO), 15 arc-second interval, 43,200 × 86,400 Gridded Dataset	2023	GEBCO Compilation Group (2023) GEBCO 2023 Grid (10.5285/f98b053b-0cbc-6c23-e053-6c86abc0af7b)	^38^

## Methods

The Caribbean is collection of islands and 3 land locked states bordered by the Caribbean Sea. The Caribbean Sea is a semi-enclosed sea defined as the water body bounded by 8^o^N–22°N latitude and 60^o^W–89^o^W longitude^7^. The paper is not solely focused on the Caribbean Sea but on the area of the ocean which interacts with Caribbean territories. This area of ocean though primarily the Caribbean Sea, also includes a small section of the North Atlantic. Therefore, in this paper an extended Caribbean Region (5^o^N–30° N Latitude and 59^o^W–90^o^W Longitude) is investigated. The extension to 30^o^N is done to capture the islands of the Bahamas which span up to above 27^o^N, while the slight westward and eastward extensions capture Belize (88^o^W) and Barbados (59^o^W) respectively.

The equation of state to calculate the sea-level change due to steric changes was obtained from the Thermodynamic Equation of State of Seawater (TEOS-10)^20,72,73^. In order to complete this calculation, the temperatures were converted to conservative temperatures and the salinity to absolute salinity as done in Meli et al.^20^, using the Gibbs Seawater Oceanographic Toolbox^74^. The absolute salinity and conservative temperature were then used to calculate the density within the water column (Roquet et al.^75^) ,. The density anomalies were determined with respect to a reference density and these anomalies integrated across the upper 900 m to arrive at the steric as outlined in the equation below.

$${\varvec{H}}{\text{steric}}=\underset{-900}{\overset{0}{\int }}\frac{{\varvec{\rho}}{\varvec{o}}\left({\varvec{S}}{\varvec{o}},{\varvec{T}}{\varvec{o}},{\varvec{P}}{\varvec{o}}\right)-\boldsymbol{ }{\varvec{\rho}}\left({\varvec{S}},{\varvec{T}},{\varvec{P}}\right)}{{\varvec{\rho}}{\varvec{o}}\left({\varvec{S}}{\varvec{o}},{\varvec{T}}{\varvec{o}},{\varvec{P}}{\varvec{o}}\right)}\boldsymbol{ }{\varvec{d}}{\varvec{z}}$$

H_steric_ is the steric height, ρ_o_ is the reference density, S_o_ the reference salinity, T_o_ the reference pressure, P_o_ the reference pressure and ρ represents the density of the sea water which varies. The reference salinity, temperature and pressure are the time mean salinity, temperature and pressure from 1993 to 2019. Steric sea-level changes can be further separated into the thermosteric and halosteric components^22,76^. Thermosteric sea-level changes are the steric changes associated with the expansion and contraction of water with temperature changes hence leading to increases and decreases in volume respectively. It was calculated using equation below:

$${\varvec{H}}{\text{thermosteric}}=\underset{-900}{\overset{0}{\int }}\frac{{\varvec{\rho}}{\varvec{o}}\left({\varvec{S}}{\varvec{o}},{\varvec{T}}{\varvec{o}},{\varvec{P}}{\varvec{o}}\right)-\boldsymbol{ }{\varvec{\rho}}\left({\varvec{S}}{\varvec{o}},{\varvec{T}},{\varvec{P}}\right)}{{\varvec{\rho}}{\varvec{o}}\left({\varvec{S}}{\varvec{o}},{\varvec{T}}{\varvec{o}},{\varvec{P}}{\varvec{o}}\right)}\boldsymbol{ }{\varvec{d}}{\varvec{z}}$$

Here salinity is held constant at the reference absolute salinity therefore the changes in steric height are solely due to the temperature variability^22^. The halosteric component was calculated using :

$${\varvec{H}}{\text{halosteric}}=\underset{-900}{\overset{0}{\int }}\frac{{\varvec{\rho}}{\varvec{o}}\left({\varvec{S}}{\varvec{o}},{\varvec{T}}{\varvec{o}},{\varvec{P}}{\varvec{o}}\right)-\boldsymbol{ }{\varvec{\rho}}\left({\varvec{S}},{\varvec{T}}{\varvec{o}},{\varvec{P}}\right)}{{\varvec{\rho}}{\varvec{o}}\left({\varvec{S}}{\varvec{o}},{\varvec{T}}{\varvec{o}},{\varvec{P}}{\varvec{o}}\right)}\boldsymbol{ }{\varvec{d}}{\varvec{z}}$$

Here temperature is held constant at the reference conservative temperature therefore the changes in steric height are solely due to variability in salinity^76^. The upper 900 m of the profiles was used to assess steric changes as this layer has been shown to be most important for steric changes in the Caribbean^13,14^. The linear trend was obtained using a linear least-square fitting^10^. The non-parametric Mann–Kendall test^77,78^ which was modified to account for the autocorrelation in the data^20,79^ was used to verify the statistical significance of the trends in in the study at the 95% confidence level. The statistically significant difference in trends was also confirmed using a two-tailed student t-test^17^.

Change point analysis through binary-segmentation analysis^20,47,48^ (see (Meli et al.^20^), for further details) is applied to the altimetry and steric time series. This method uses a radial bias function to approximate change point in non-parametric timeseries. This method allows for identification of unevenly spaced changepoints which makes it ideal for use in sea level timeseries analysis. Sensitivity analysis of the trends was done^80^ for the trends calculated for the different periods.

## Data Availability

All data is free to access and is available at the URLs outlined in Table 1.
